# Supplementary material for: Super-Long SERS Active Single Silver Nanowires for Molecular Imaging in 2D and 3D Cell Culture Models
Source: Biosensors (Basel). 2022 Oct 15;12(10):875. doi: 10.3390/bios12100875 (PMC9599576; doi:10.3390/bios12100875)
Supplement: Supplementary file 1 [file biosensors-12-00875-s001.zip › biosensors-1957322-supplementary.pdf]

biosensors

Supplementary Information

Super-Long SERS Active Single Silver Nanowires for Molecular Imaging in 2D and 3D Cell Culture Models

Xiao-Tong Pan [1], Xuan-Ye Yang [2], Tian-Qi Mao [1], Kang Liu [1], Zao-Zao Chen [3], Li-Na Ji [4,*], Dechen Jiang [1,*], Kang Wang [1,*], Zhong-Ze Gu [3] and Xing-Hua Xia [1]

1 State Key Laboratory of Analytical Chemistry for Life Science, School of Chemistry and Chemical Engineering, Nanjing University, Nanjing 210023, China
2 Institute of Theoretical and Computational Chemistry, Key Laboratory of Mesoscopic Chemistry of the Ministry of Education (MOE), School of Chemistry and Chemical Engineering, Nanjing University, Nanjing 210023, China
3 State Key Laboratory of Bioelectronics, School of Biological Science and Medical Engineering, Southeast University, Nanjing 210096, China
4 State Key Laboratory of Pharmaceutical Biotechnology, School of Life Sciences, Nanjing University, Nanjing 210023, China
* Correspondence: jilina@nju.edu.cn (L.-N.J.); dechenjiang@nju.edu.cn (D.J.); wangkang@nju.edu.cn (K.W.)

1. Fabrication of Carbon Nanoelectrodes by Chemical Vapor Deposition (CVD).

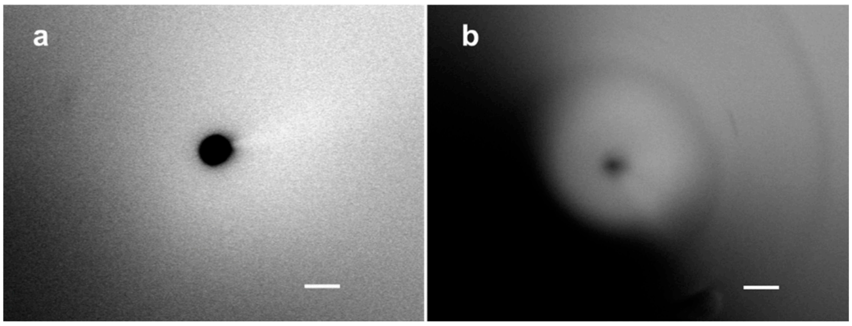

Figure S1. SEM images of the glass capillary orifice before (a) and after (b) CVD. Scale bars in (a) and (b): 100 nm.

We used carbon nanoelectrodes with a diameter of 40 nm as reported previously[1]. The present work shows that carbon nanoelectrodes with a diameter of 100 nm could also be used in the growth of AgNWs.

2. Effect of Reduction Current on the Surface Roughness of AgNW.

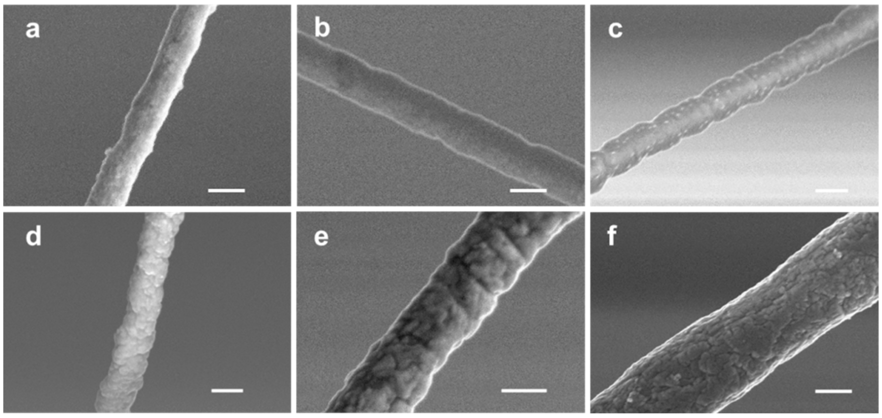

Figure S2. SEM images of AgNWs synthesized by reduction currents of 0.2 nA (a), 0.5 nA (b), 1 nA (c), 1.5 nA (d), 2 nA (e) and 2.5 nA (f), respectively. Scale bars: 200 nm. As observed, the diameter and roughness of AgNWs increases with the reduction current.

The range of reduction current for AgNW growing was 0.7~1.2 nA in our previous report[1], whileit was expanded to 0.2~2.8 nA in the present study. The change in reduction current range might be due to the reasonthat the potentiostat was changed from Autolab to CHI 660E.

3. Evaluation of Raman Enhancement.

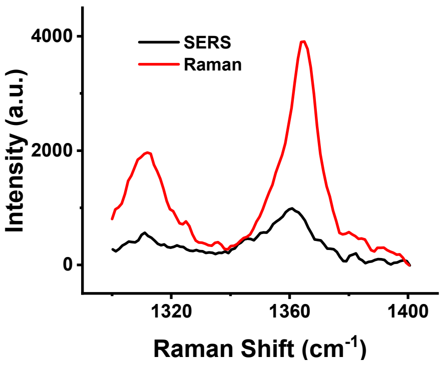

Figure S3. SERS spectrum (black) of 10^{-9} M Rhodamine (R6G) on an AgNW and Raman spectrum (red) of 10^{-3} M R6G.

The SERS spectrum was taken from an AgNW immersed in 10^{-9} M R6G solution under 20 s integration time. The Raman spectrum of 10^{-3} M R6G was also measured under 20 s integration time.

The Raman enhancement factor (EF) was estimated according to the following Equation **S1**:

$$\mathrm{EF} = \frac{I_{SERS}/N_{SERS}}{I_{Raman}/N_{Raman}} = \frac{I_{SERS}/C_{SERS}}{I_{Raman}/C_{Raman}} \qquad \textbf{(S1)}$$

I_{SERS} and I_{Raman} indicate the intensities of the specific Raman bands. Herein, the band around 1362 cm^{-1} is selected for EF calculation. N_{SERS} is the number of molecules contributing to I_{SERS} while N_{Raman} is the number of molecules that yield I_{Raman}. C_{SERS} and C_{Raman} indicate the concentration of solutions used in SERS and Raman measurement, respectively.

As shown in **Figure S3**, I_{SERS} is around 988 and I_{Raman} is around 3909, while C_{SERS} is 10^{-9} M and C_{Raman} is 10^{-3} M.

Thus, the EF is calculated to be:

EF = 2.53×10^5

4. Relative Standard Deviation (RSD) of Band Area on AgNW

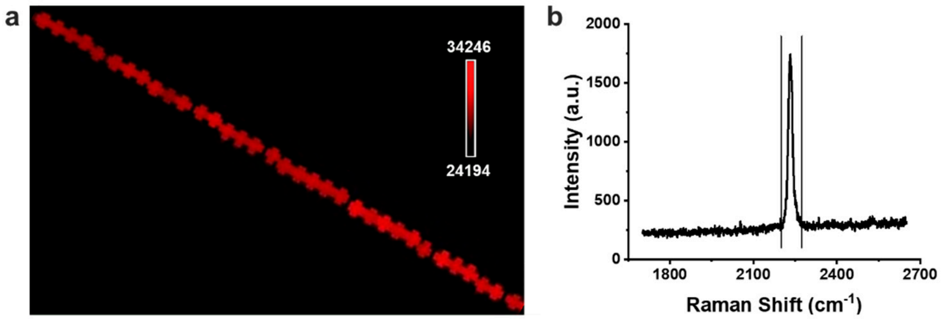

Figure S4. (a) SERS mapping of an AgNW modified with 4-MBN. Data points were taken every 1 μm with the laser power of 10% and the spectra integration time of 2 s. (b) One of the typical spectra in the SERS mapping. The band area between 2200 cm^{-1} and 2274 cm^{-1} in the spectra was integrated for RSD calculation.

Table S1. The SERS band areas of cyano group along an AgNW.

Rank	Band Area (a.u.)	Rank	Band Area (a.u.)
1	29143.195	18	29195.261
2	28961.739	19	29647.917
3	28054.444	20	30213.038
4	28271.406	21	29644.97
5	28078.107	22	29849.891
6	28673.71	23	31913.219
7	29944.632	24	30568.047
8<br>8	29326.627	25	32207.109
9	29450.894	26	30575.937
10	27439.081	27	31211.052
11	29746.752	28	29060.356
12	29468.646	29	32615.391
13	30752.663	30	31282.051
14	30095.683	31	31857.992
15	29380.558	32	30761.382
16	29482.24	33	32082.844<br>30481.278
17	29502.959	34	30481.278

The RSD of the band areas of cyano group along the AgNW is calculated according to the following equation **S2**:

$$RSD = \frac{\sqrt{\sum_{i=1}^{n}(x_i - \bar{x})^2}}{\bar{x}} \times 100\% \qquad \textbf{(S2)}$$

where $\bar{x}$ is the average value of the band area. Thus, the RSD is calculated to be 4.22%.

5. Contour Graphs of Raman Spectra of Cell Culture Models.

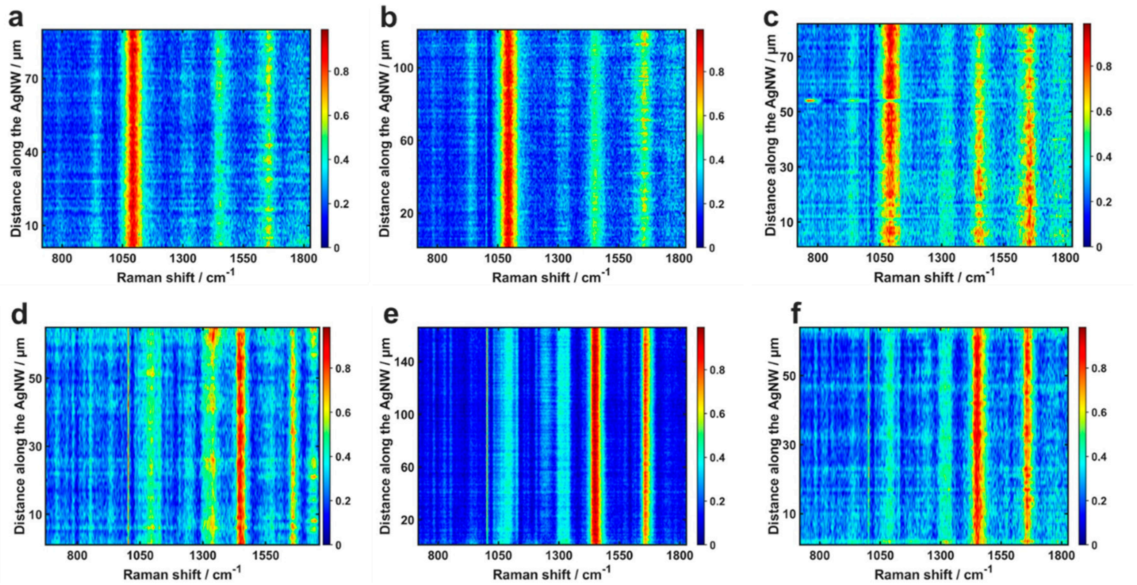

Figure S5. Contour graphs of Raman spectra along AgNW in 2D (a-c) and 3D (d-f) cell culture models. The number of SERS spectra obtained in the contour graphs was 90 (a), 121(b), 82(c), 65(d), 166(e), and 64(f), respectively.

6. Schematic Diagram of the Random Forest-Combined K-means++ Algorithm.

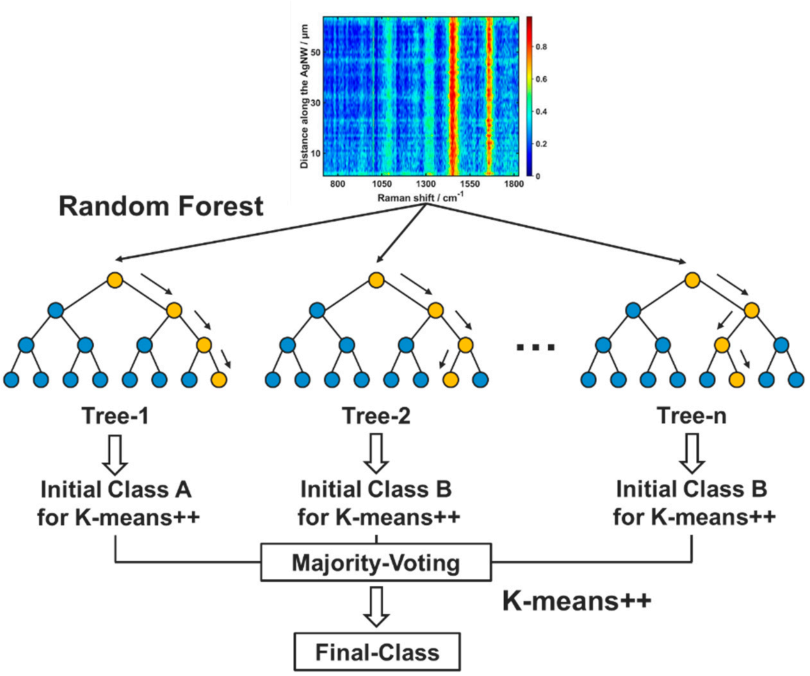

Figure S6. Schematic diagram of the Random Forest-combined K-means++ algorithm.

7. Application of Random Forest in 3D Model.

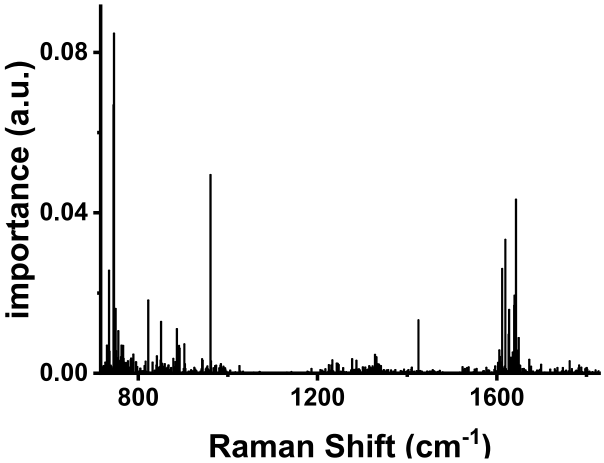

Figure S7. Importance of Raman shift learned from 562 SERS spectra of 3D models using Random Forest algorithm.

References

1. Pan, X.-T.; Liu, Y.-Y.; Qian, S.-Q.; Yang, J.-M.; Li, Y.; Gao, J.; Liu, C.-G.; Wang, K.; Xia, X.-H., Free-standing single Ag nanowires for multifunctional optical probes. *ACS Appl. Mater. Inter.* **2021,** *13,* 19023–19030.
